# The added value of whole-body magnetic resonance imaging in the management of patients with advanced breast cancer

**DOI:** 10.1371/journal.pone.0205251

**Published:** 2018-10-12

**Authors:** Fabio Zugni, Francesca Ruju, Paola Pricolo, Sarah Alessi, Monica Iorfida, Marco Angelo Colleoni, Massimo Bellomi, Giuseppe Petralia

**Affiliations:** 1 Post-graduation school in Radiodiagnostics, University of Milan, Milan, Italy; 2 Department of Radiological Science and Radiation Therapy, European Institute of Oncology (IEO), Milan, Italy; 3 Division of Medical Senology, European Institute of Oncology (IEO), Milan, Italy; 4 Department of Oncology, University of Milan, Milan, Italy; University of South Alabama Mitchell Cancer Institute, UNITED STATES

## Abstract

This study investigates the impact of whole-body MRI (WB-MRI) in addition to CT of chest-abdomen-pelvis (CT-CAP) and 18F-FDG PET/CT (PET/CT) on systemic treatment decisions in standard clinical practice for patients with advanced breast cancer (ABC). WB-MRI examinations in ABC patients were extracted from our WB-MRI registry (2009–2017). Patients under systemic treatment who underwent WB-MRI and a control examination (CT-CAP or PET/CT) were included. Data regarding progressive disease (PD) reported either on WB-MRI or on the control examinations were collected. Data regarding eventual change in treatment after the imaging evaluation were collected. It was finally evaluated whether the detection of PD by any of the two modalities had induced a change in treatment. Among 910 WB-MRI examinations in ABC patients, 58 had a paired control examination (16 CT-CAP and 42 PET/CT) and were analysed. In 23/58 paired examinations, additional sites of disease were reported only on WB-MRI and not on the control examination. In 17/28 paired examinations, PD was reported only on WB-MRI and not on the control examination. In 14 out of the 28 pairs of examinations that were followed by a change in treatment, PD had been reported only on WBMRI (14/28; 50%), while stable disease had been reported on the control examination.

In conclusion, WB-MRI disclosed PD earlier than the control examination (CT-CAP or PET/CT), and it was responsible alone for 50% of all changes in treatment.

## Introduction

Advanced breast cancer (ABC) encompasses metastatic (MBC) and locally advanced breast cancer (LABC). MBC is still an incurable disease with a 5-year survival rate between 15% and 27% [1,2], and progression to MBC occurs in about 20–30% of non-metastatic patients [3]. LABC is diagnosed at presentation in 8.5% of American and 4% of European patients with breast cancer, and despite aggressive treatment, many of these patients eventually progress to MBC, with a 5-years survival rate between 15% and 50% [1]. Once the diagnosis if ABC is made, a systemic antineoplastic treatment is offered, including endocrine therapy, chemotherapy or targeted therapy. The most common site of metastases is bone, with more than 70% of those who die from breast cancer having evidence of bone metastases [4], followed by liver, pleura, peritoneum, lung, distant lymph nodes and central nervous system [5].

In addition, a recent meta-analysis including 127,324 patients demonstrated that during a 5-years follow-up a median of 12.2% of non-metastatic breast cancer patients (stages I-III) will develop bone metastases [6]. Bone disease can impact on quality of life, causing skeletal related events (SREs): with significant health economic implications [7].

Recognizing metastatic disease progression promptly and confidently is crucial for ABC management, since it allows for a timely initiation of a new line of therapy. Evaluation of response to treatment in ABC patients can be performed using several imaging modalities [8]. The most widely available is computerised tomography (CT) which provides a good compromise between quickness, feasibility, cost-effectiveness and reproducibility of the examination. Evaluation of disease response or progression on CT relies mainly on morphologic modifications; the Response Evaluation Criteria In Solid Tumours (RECIST) version 1.1 are the most widely accepted for this purpose [9]. However, CT shows limitations in the evaluation of patients with bone-predominant MBC. In fact, RECIST consider bone metastases to be non-measurable [9]. The MD Anderson Cancer Centre criteria [10] have provided other CT features for defining sclerotic response to treatment and progression. Unfortunately, in patients that show heterogeneous response to treatment and in those who have bone only disease, these criteria cannot be applied. Moreover, these criteria are not valid in patients who receive anti-osteoclastic therapy (bisphosphonates). ^18^F-Fluorodeoxyglucose positron emission tomography (PET/CT) is a functional imaging modality that depicts the intensity of glucose metabolism across different body regions. This feature allows PET/CT to assess disease response or progression by revealing changes in metabolic activity within neoplastic tissue even without the evidence of morphological changes. Despite the overall better diagnostic performance of PET/CT compared to CT and bone scintigraphy, these two remain the most widely recommended in the follow-up of ABC patients [11]. This is also due to differences in availability but, more importantly, to the absence at present of a clear advantage in terms of cost-benefit ratio. The European School of Oncology–Metastatic Breast Cancer (ESO-MBC) Task Force guidelines state that PET/CT is not recommended for routine restaging of MBC patients. “Alternative” radiotracers might play a role in the future for the follow-up and response-evaluation of MBC patients in specific settings, as in the case of tumour heterogeneity. ^89^Zr-trastuzumab has been used for the detection of human epidermal growth factor 2 (HER2)-positive metastases developed in patients with HER2-negative tumours [12]; ^18^F-Fluciclovine uptake has shown good correlation with treatment response to neoadjuvant systemic treatments [13], as well as a higher sensitivity for infiltrating lobular carcinoma (ILC) [14]. Whole-body PET/MRI is a hybrid imaging modality that merges the advantages of both techniques. Although ideally suitable for the long term surveillance of cancer survivors due to the reduced radiation dose compared to PET/CT [15], current studies are mainly focusing on its use for disease staging [16] and response evaluation after neoadjuvant treatments [17]. At present, the true added value of simultaneous PET/MRI, as compared to PET/CT and MRI, remains to be determined [15].

Whole-body magnetic resonance imaging (WB-MRI) is emerging as a promising bone marrow assessment tool for detection and therapy monitoring of bone metastases in different tumour types [18–20].WB-MRI features T1 and T2 sequences for morphologic evaluation, as well as diffusion-weighted imaging (DWI) which highlights areas of reduced water diffusivity within tissues. Previous publications have provided criteria for the use of WB-MRI for therapy monitoring of MBC patients [20,21], which rely on DWI as indirect but solid biomarker of tissue cellularity [22]. DWI enables the radiologist to rapidly recognize the presence and evolution of metastatic disease, providing early signs of disease response or progression [21]. Apparent Diffusion Coefficient (ADC) maps derive from a quantitative analysis of diffusion-weighted images; ADC values show an inverse correlation with cellularity in many tumour histotypes, including breast cancer [23]. Evaluating the findings highlighted by DWI according to their corresponding ADC values helps differentiating benign from malignant lesions [24]. Notably, DWI alone is not always accurate this task [25], therefore correlation with morphologic T1 and T2 images is mandatory for avoiding false-positive and false-negative results [26]. One of the strengths of WB-MRI is that it can be performed in clinically acceptable examination times (30–40 min). Two other great advantages include the lack of contrast injection and ionising radiation. Due to the superior sensitivity for initial bone infiltration, WB-MRI has become the gold standard in the diagnosis and assessment of multiple myeloma [27,28]. Guidelines for acquisition, interpretation and reporting of WB-MRI in advanced prostate cancer [29] have recently been published, as a support for the increasing use of WB-MRI in metastatic and high-risk prostate cancer. Due to high performance in bone-metastatic tumours, WB-MRI has been naturally suggested for the evaluation of MBC. At our centre, an increasing number of patients with bone-predominant or bone-only MBC is being evaluated using WB-MRI, alongside other whole-body imaging modalities such as CT-CAP and PET/CT. Two imaging modalities may occasionally be performed at the same time point for the characterization of complex metastatic patterns, unclear tumour behaviour, or when the hypothesis of disease progression is affected by discordant data.

This retrospective study of our practice in the evaluation of ABC patients under systemic treatment investigates whether the use of WB-MRI in addition to chest-abdomen-pelvis CT (CT-CAP) or PET/CT brings an additional benefit in patient management, allowing for earlier changes of ineffective treatments.

## Methods and materials

### Population

We interrogated an ongoing registry including all WB-MRI performed in our institute from February 1^st^ 2009 to April 31^st^ 2017 (2444 examinations). We included in the study all WB-MRI performed on ABC patients undergoing systemic treatment; all examinations performed for baseline disease staging were excluded. All WB-MRI performed within 8 weeks from a CT-CAP or an ^18^F-FDG PET/CT were included in the study. Each WB-MRI included in the study was therefore paired to a CT-CAP or a PET/CT (defined as “control examinations”) performed under the same systemic treatment. Patient’s medical records were made available for all included examinations; more specifically, we collected the medical records compiled at the start of the ongoing treatments, and those compiled immediately after each pair of examinations (within one month). The ethics committee of our institution (European Institute of Oncology, Milan) approved this study. The ethics committee waived the requirement for informed consent, as this retrospective study did not alter or influence the diagnostic and therapeutic paths of the patients, involving summary data from imaging reports and medical records.

### Imaging technique

Our WB-MRI protocol Table 1, consisting of sagittal T1-weighted and T2-weighted sequences on the whole spine, axial T1 weighted, T2-weighted and DWI from head to mid-thigh, was performed on a 1.5 T scanner (Magnetom Avanto, Siemens Healthcare Sector, Erlangen, Germany). Anatomy-specific phased-array surface coils were used for all body regions. The typical cumulative WB-MRI data acquisition time was 40 minutes. Post-processing included in-line (Water e Fat images, ADC maps) and off-line reconstruction (Radial maximum intensity projections of high b-value images, multi-plane reconstructions and relative fat-fraction maps). CT-CAP and ^18^FDG PET-CT were performed using each imaging department’s standard imaging protocols. CT-CAP always included intravenous contrast administration. All the original WB-MRI scans were reported by a pool of three radiologists (one senior radiologist and two junior radiologists who were supervised by the senior one, with an experience of 8, 5 and 4 years respectively in oncological WB-MRI). As to the occurrence of stable or progressive disease, particularly in the skeleton, images were interpreted according to the criteria proposed by Padhani et al. [21].

**Table 1:** Scanning parameters for WB-MRI.

Scanning parameters (1,5T)	Chest—Abdomen—Pelvis		Spine		DWI
Image Contrast	T1	T2	T1	T2	DWI
	In/Out-Phase				
Imaging Sequence	DIXON	HASTE	TSE	TSE	SSH SE EPI^a^
Orientation	Axial	Axial	Sagittal	Sagittal	axial
Echo / Repetition Time (ms)	2.39–4.77 / 6.65	74 / 800	9.3 / 350	60 / 2560	62 / 6550
Field of view (mm)	430	430	400	400	430
Matrix	352 x 209	320 x 175	448 x 224	320 x 160	132 x 120
Slices per Station / Stations	72 / 4	176 / 1	16 / 2	16 / 2	50 / 4
Flip angle (degrees)	20.5	149	150	150	-
Slice Thickness / Gap (mm)	3.5 / 0.7	5 /1	4 / 0.4	4 / 0.4	5 / 0
Fat Suppression	…	…	…	STIR	STIR
Respiratory Control	Breath-Hold	Breath-Hold	…	…	Free-breathing
Diffusion Encoding: b-values (s/mm2)	…	…	…	…	50, 900
Acquisition Time (min:sec)	1:04	2:30	3:32	3:10	15:02

^a^ Single-Shot Spin Echo Planar Imaging

### Data collection

Two residents in radiology with 3 and 1 years’ experience in WB-MRI reporting independently extracted the relevant data from the original reports of WB-MRI and the control examinations. Firstly, data regarding the reported extent of disease were collected, annotating the presence or absence of metastases categorized by 7 sites: bone, primary site, lymph nodes (including regional and distant), liver, lung/pleura, peritoneum/retroperitoneum (including stomach, bowel, intra and retro-peritoneal fat), other.

Sites of metastatic disease reported by only one of the two modalities (either WB-MRI or the control examination) were annotated and termed additional sites (AS). Discordance on the assessment of disease extent was defined as the presence of one or more AS reported by one of the two modalities.

Secondly, reports were reviewed to determine whether WB-MRI and/or the control examination had described progression of disease (PD), defined as the increased extent of the disease compared to the patient’s known baseline at the start of the ongoing treatment. Discordance on reported PD was defined as occurring when WB-MRI and the control examination reports concluded with different assessments (i.e. PD opposed to stable disease). When only one modality described PD, the sites of disease in which PD was reported were annotated.

Thirdly, data were collected regarding all cases in which the ongoing treatment was changed as result of the imaging response. In all cases of discordance between the two modalities, data were collected as to whether the PD reported by one of the two examinations had motivated a change in treatment, as stated in each patient’s medical record.

## Results

A total of 910 WB-MRI examinations were performed on ABC patients in the selected period. Fifty-eight WB-MRI were paired to a control examination: 16 (28%) to CT-CAP and 42 (72%) to PET/CT. The median age of the included patients was 56 years (range, 36–80 years). Information on the characteristics of the breast cancers in these patients is summarized in Table 2. The median time distance between WB-MRI and the control examination was 27 days (range, 1–54 days). In 40 pairs of examinations (69%) the status of the patient at the time of the evaluation was M1, while in 18 pairs (31%) it was M0 (no prior history of metastases).

**Table 2:** Breast cancer characteristics in our study population.

	N	%
**Histology**		
IDC	37	64%
ILC	19	32%
Other	1	2%
Unknown	1	2%
**Grade**		
1	2	3%
2	23	40%
3	18	31%
Unknown	15	26%
**ER**		
Positive	54	93%
Negative	4	7%
**PgR**		
Positive	53	91%
Negative	5	9%
**HER2**		
Positive	31	53%
Negative	24	41%
Unknown	3	5%
**M**		
Positive	40	69%
Negative	18	31%

Abbreviations: IDC = invasive ductal carcinoma; ILC = invasive lobular carcinoma; ER = oestrogen receptor; HER2 = human epidermal growth factor 2; M = metastatic status.

### Extent of disease

Out of the 58 paired examinations, metastases were reported in 49 WB-MRI and in 39 control examinations. Bone metastases were the most frequent, reported by 37 WB-MRI and by 30 control examinations. The second most frequent site of metastases was lymph nodes, reported in 15 WB-MRI and in 18 control examinations. Discordant assessments on disease extent were observed in 28 paired examinations. AS were reported in 23 WB-MRI and in five control examinations, all of which were PET/CT. The distribution of the AS is summarized in Table 3.

**Table 3:** Distribution of the additional sites of disease (AS) reported by WB-MRI and by the control examination respectively.

AS in WB-MRI				AS in the control examination (PET/CT)			
8	bone only			0	bone only		
5	bone + visceral/soft tissues	1	Liver	1	bone + visceral/soft tissues	1	lung/pleura
		1	Liver + lymph nodes				
		2	Lymph nodes				
		1	Peritoneum/retroperitoneum				
10	Visceral/soft tissues	5	Peritoneum/retroperitoneum	4	Visceral/soft tissues	3	Lymph nodes
		2	Lung/pleura				
		1	Liver				
		1	Local			1	Peritoneum/retroperitoneum
		1	Other				
23	Total			5	Total		

### Assessment of progressive disease

Of the 58 pairs of examinations, SD was reported in 18 pairs and PD in a total of 40 pairs. PD was reported by both examinations in 23 out of 40 pairs, while in the other 17 it was only reported by WB-MRI, with the control examination reporting SD. Table 4 summarizes the anatomical distribution of the sites of PD detected only by WB-MRI and of those detected by both examinations. Twelve out of 18 non-metastatic (M0) patients were up-staged to M1, in seven of these, PD was reported only on WB/MRI.

**Table 4:** Distribution of the sites of progressive disease (PD) reported only at WB-MRI and of those reported at both examinations.

PD reported by both examinations in 23 pairs				PD reported only by WB-MRI in 17 pairs				PD reported only by the control examination in 0 pairs	
9	Bone			8	Bone			0	Bone
12	Bone + visceral/soft tissues	3	Lymph nodes	4	Bone + visceral/soft tissues	2	Lymph nodes	0	Bone + visceral/soft tissues
		2	Lymph nodes + liver						
		1	Lymph nodes + lung/pleura			1	Limph nodes + liver		
		1	Lymph nodes + other						
		2	Lung/pleura						
		2	Peritoneum/retroperitoneum			1	Peritoneum/retroperitoneum		
		1	Liver						
2	Visceral/soft tissues	1	Lymph nodes	5	Visceral/soft tissues	3	Peritoneum/retroperitoneum	0	Visceral/soft tissues
						1	Lung/pleura		
		1	Lymph nodes + lung/pleura			1	Local		

### Change in treatment

**Fig 1:**
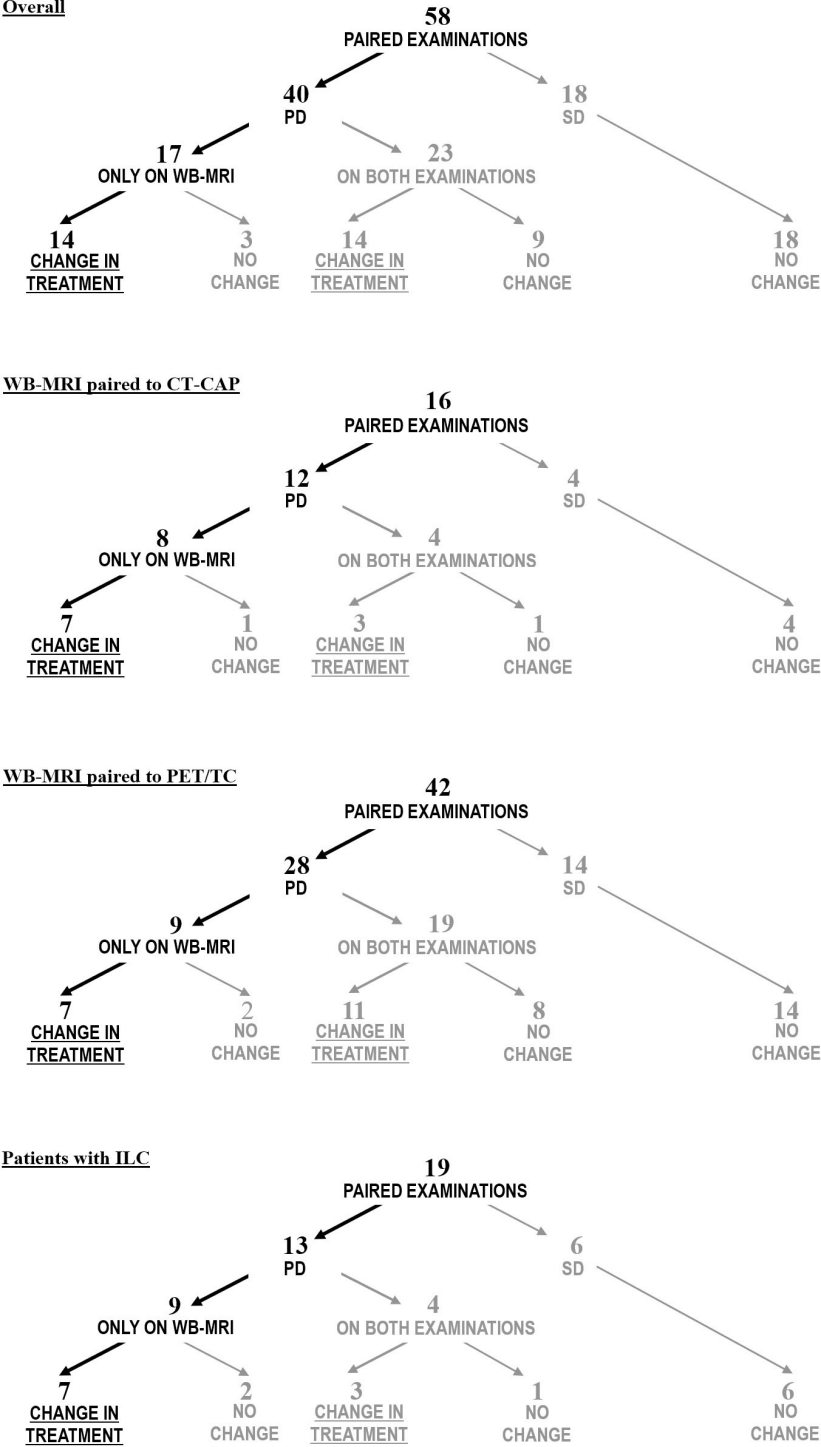
Impact on treatment: Overall and subgroup analyses. The flowcharts in this picture illustrate how often the imaging outcomes motivated changes of treatment in our cohort, with a distinction between the cases of PD reported only on WB-MRI and those in which PD was reported on both examinations (WB-MRI and the control examination). The first flowchart includes all 58 paired examinations (overall analysis). Separate analyses are shown for specific subgroups in the following flowcharts, including analyses of all 16 paired examinations in which WB-MRI was paired to CT-CAP, all 42 paired examinations in which WB-MRI was paired to PET/CT and all 19 paired examinations performed in ILC patients. Abbreviations: PD = progressive disease; SD = stable disease.

A change in treatment was made in 28 out of 40 pairs as a result of PD; in 12 pairs therapy was continued despite evidence of PD. Overall, treatment was changed because of PD in 28 episodes, of which 14 (50%) were due to PD reported only by WB-MRI Fig 1. Separate analyses for the paired evaluations in which WB-MRI was compared to CT-CAP and to PET/CT are graphically described in Fig 1. Inside the subgroup of 19 paired examinations in patients with ILC, therapy was changed because of PD in 10; PD was reported only on WB-MRI in seven of these (7/10; 70%) Fig 1. The most frequent AS in this subgroup was bone, being reported in seven WB-MRI, while the second was peritoneum/retroperitoneum, reported in four. Inside the subgroup of 18 paired examinations in M0 patients, therapy was changed because of PD in eight; PD was reported only on WB-MRI in six of these (6/8; 75%). Supporting information for this paper include detailed information on the extent of disease, sites of PD and impact on systemic treatment for all pairs of examinations, S1 Table.

## Discussion

### Extent of disease

**Fig 2:**
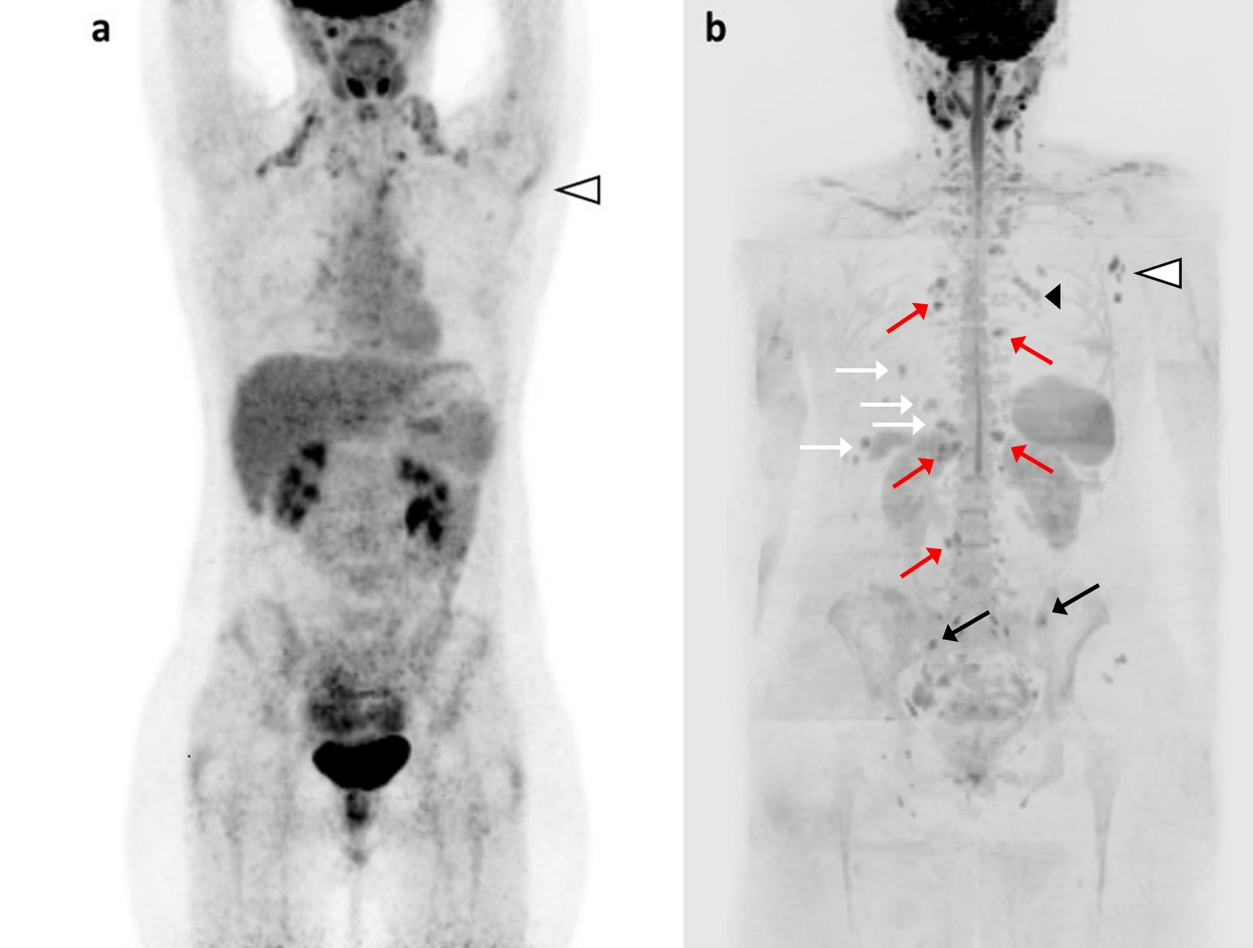
Nodal recurrence or progression to metastatic disease? 36 years old woman with locally advanced ductal breast cancer, after surgery (pT1 N1a M0), local radiation therapy and adjuvant chemotherapy. While under endocrine treatment, an axillary nodal recurrence is diagnosed (histologically proven). In the suspicion of distant metastases, the patient underwent FDG-PET/C. Coronal FDG-PET MIP (a) showed uptake in left axillary lymph nodes (white arrowhead), with no other finding suspicious for metastases. WB-MRI was performed 15 days later: DWI b-900 MIP (b) confirmed the left axillary lymph node metastases (white arrowhead), and detected metastases in bone (left iliac bone, right sacral wing; black arrows), in liver (II and VI segments; white arrows), in lymph nodes (right parasternal, left internal mammary, hepatic hilar, para-aortic and lumbar; red arrows) and in subcutaneous parasternal tissues (black arrowhead). Normal areas of high signal can be seen in cervical and pelvic lymph nodes, brain, spinal cord, spleen, kidneys and in bilateral ovarian cysts. The PD reported at WB-MRI determined a change in treatment from Letrozole to capecitabine, vinorelbine and cyclophosphamide, with additional radiation therapy on bone lesions.

**Fig 3:**
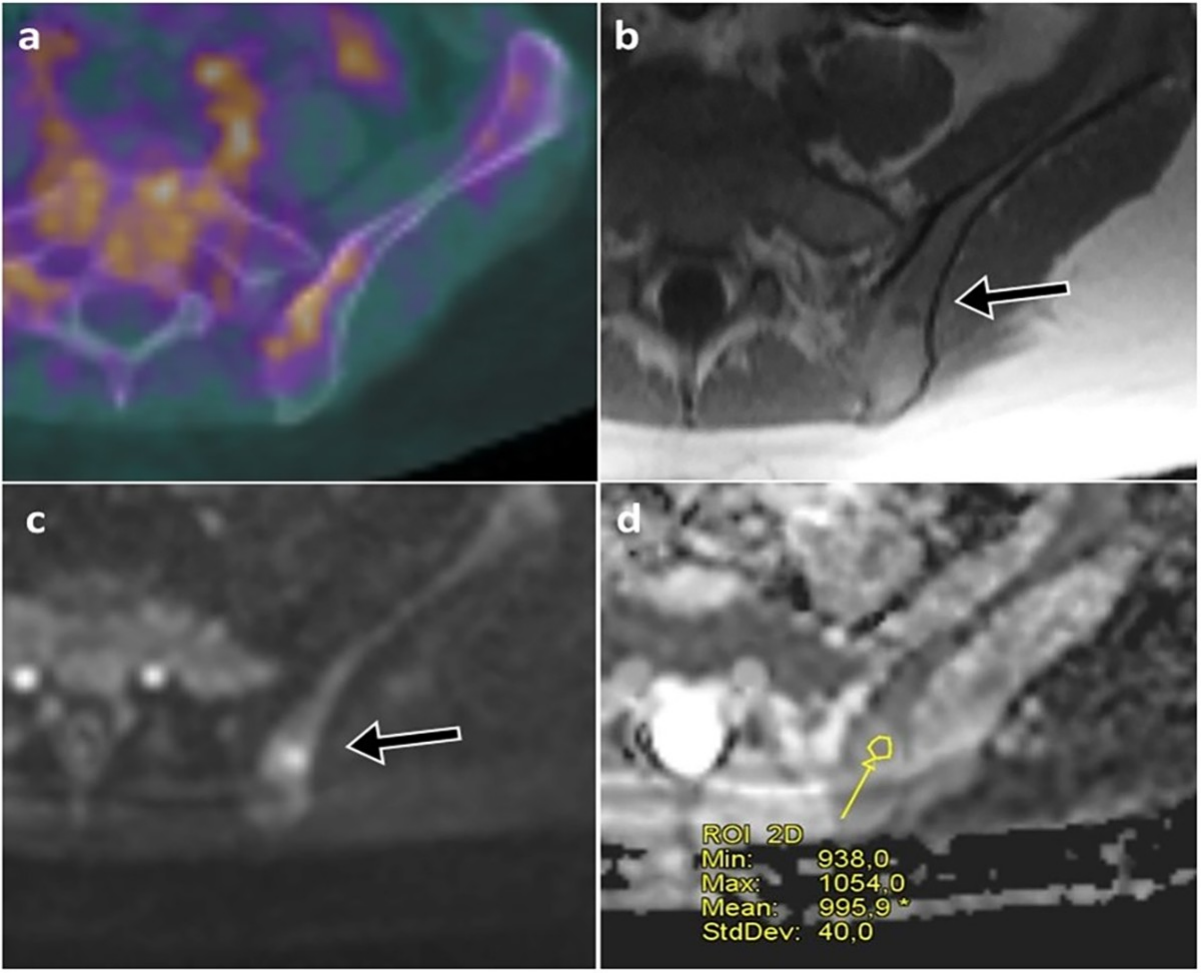
In the same pair of examinations of Fig 2, axial fused FDG-PET/CT showed non-specific FDG uptake in the pelvic bones (a). T1 weighted axial image from WB-MRI showed a suspicious bone lesion in the left iliac bone (arrow in b), which was hyperintense on b-900 DWI images (arrow in c) with low ADC values (d).

**Fig 4:**
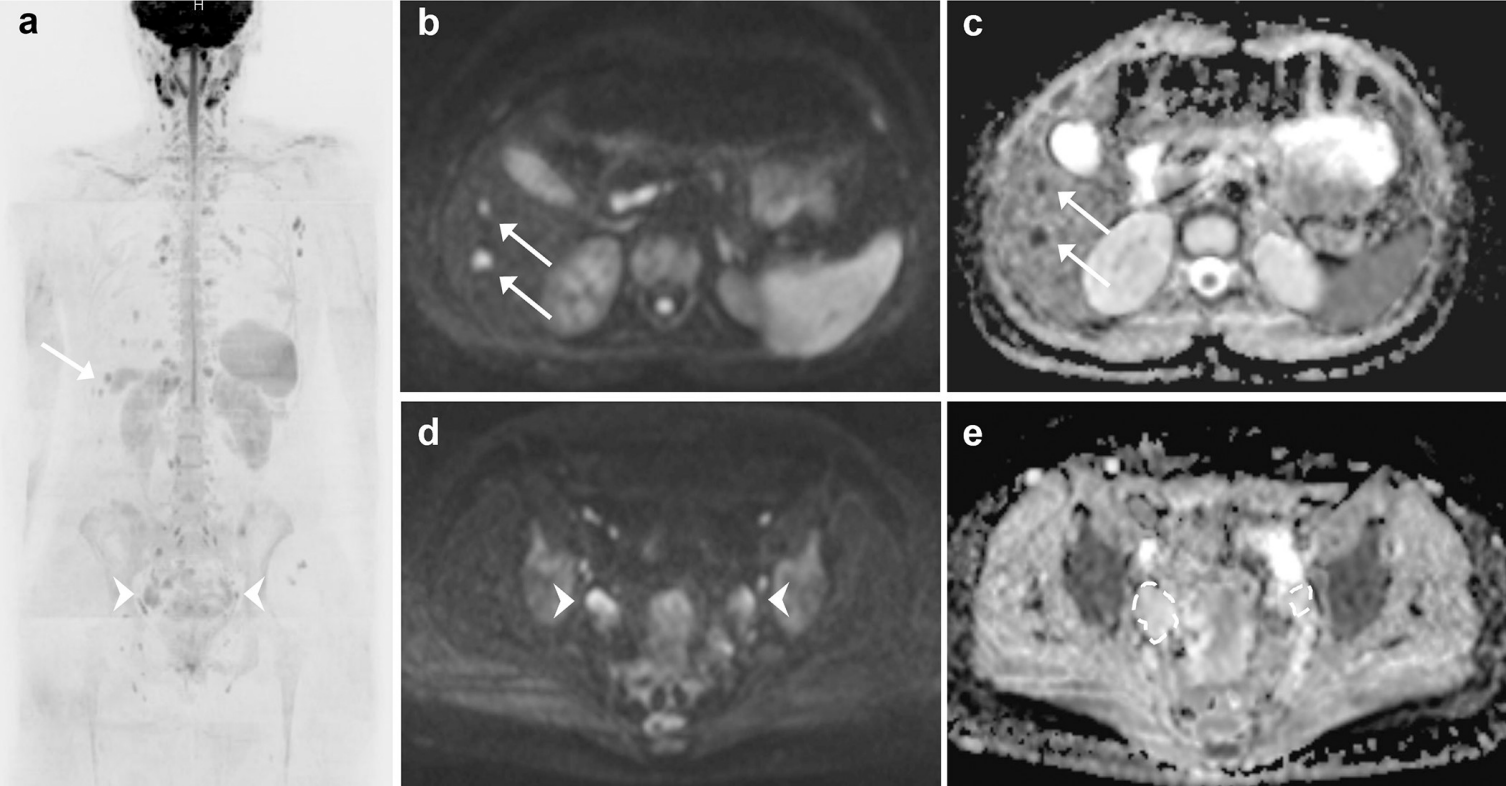
In this picture, taken from the same examination of Figs 2 and 3, two different examples of high signal intensity findings in DWI b-900 MIP (a) are illustrated. (b) B-900 DWI images reveal two new hyper intense lesions in the left liver lobe (arrows). (c) In the correspondent ADC map, the lesions (arrows) show low values, which suggest high cellularity, making them suspicious for metastases. (d) B-900 DWI images show bilateral pelvic masses with high signal intensity (arrowheads). (e) The correspondent ADC maps shows the absence of impeded diffusion within the masses, that represent follicular ovarian cysts.

As expected, in our study the most frequent site of metastatic findings in all modalities was bone, being bone the most common site of metastases in breast cancer [30]. The second and third most common sites of metastases in both modalities were lymph nodes and lung/pleura, as well mirroring the common metastatic pattern reported in literature. Bone was also the most frequent additional site (AS) of disease reported by WB-MRI and not by the control examination (Figs 2–4). A meta-analysis on the detection of bone metastases [31] has indeed reported sensitivity/specificity on a per-patient basis for MRI of 90.6% and 95.4%, proving it to be superior to CT with sensitivity/specificity of 72.9% and 94.8, and comparable to PET, with sensitivity/specificity of 89.7% (95% CI: 87.4–91.6%) and 96.8% (95% CI: 96.2–97.3%).

**Fig 5:**
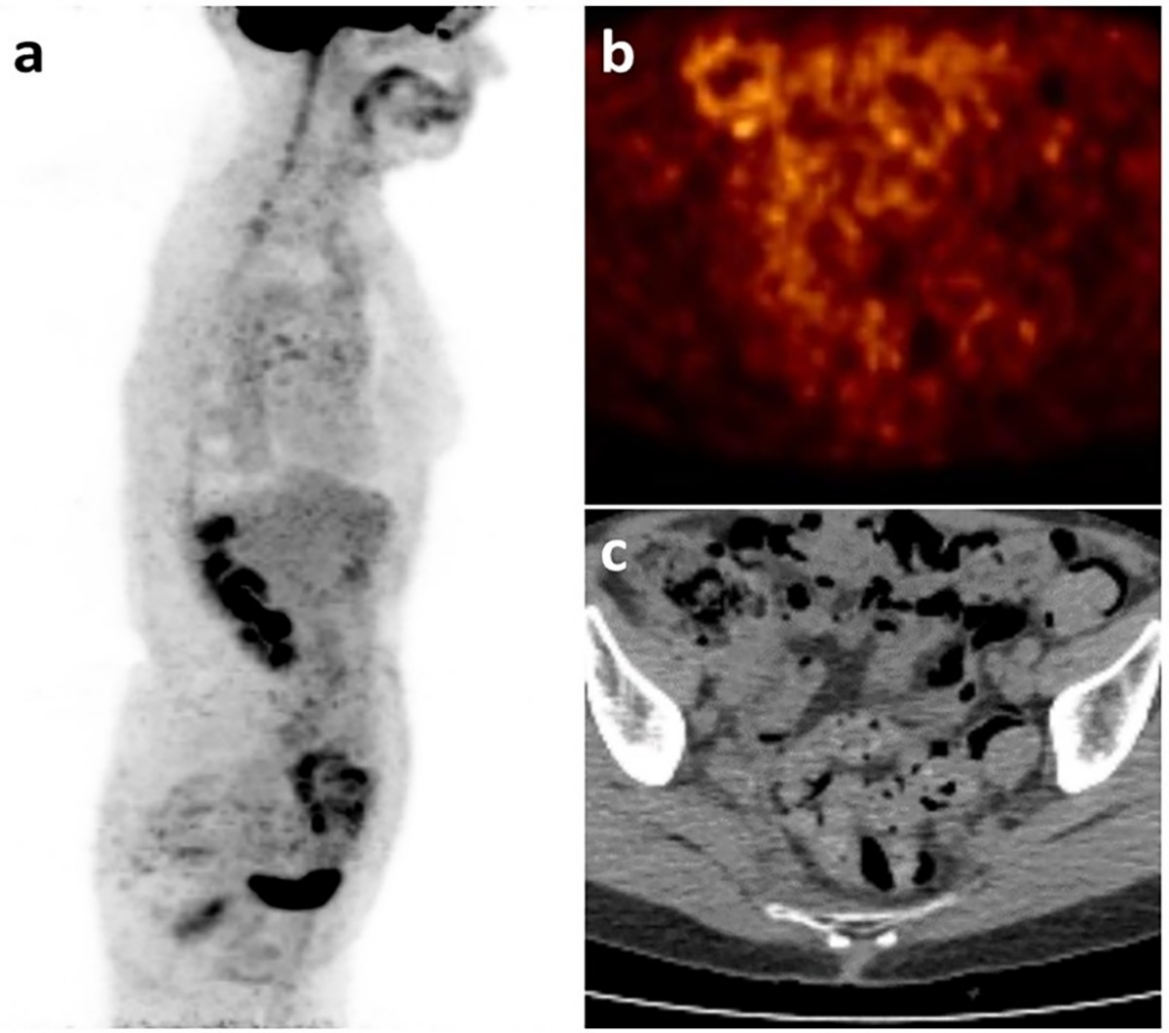
Peritoneal carcinosis undetected at PET/CT. Patient with locally advanced ductal breast carcinoma (pT2, N2a, M0) after surgery and 4 cycles of chemotherapy, under endocrine therapy. After suspicious rise in CA15-3 the patient underwent FDG-PET/CT, which showed no suspicious uptake and was reported as negative for distant metastases. Sagittal MIP (a) showed non-specific FDG uptake in the ascending colon and tracer excretion in the urinary tract. Pelvic axial cross-section of the FDG-PET/CT (b) and the co-registered CT image (c) did not show any suspicious uptake or measurable lesion.

**Fig 6:**
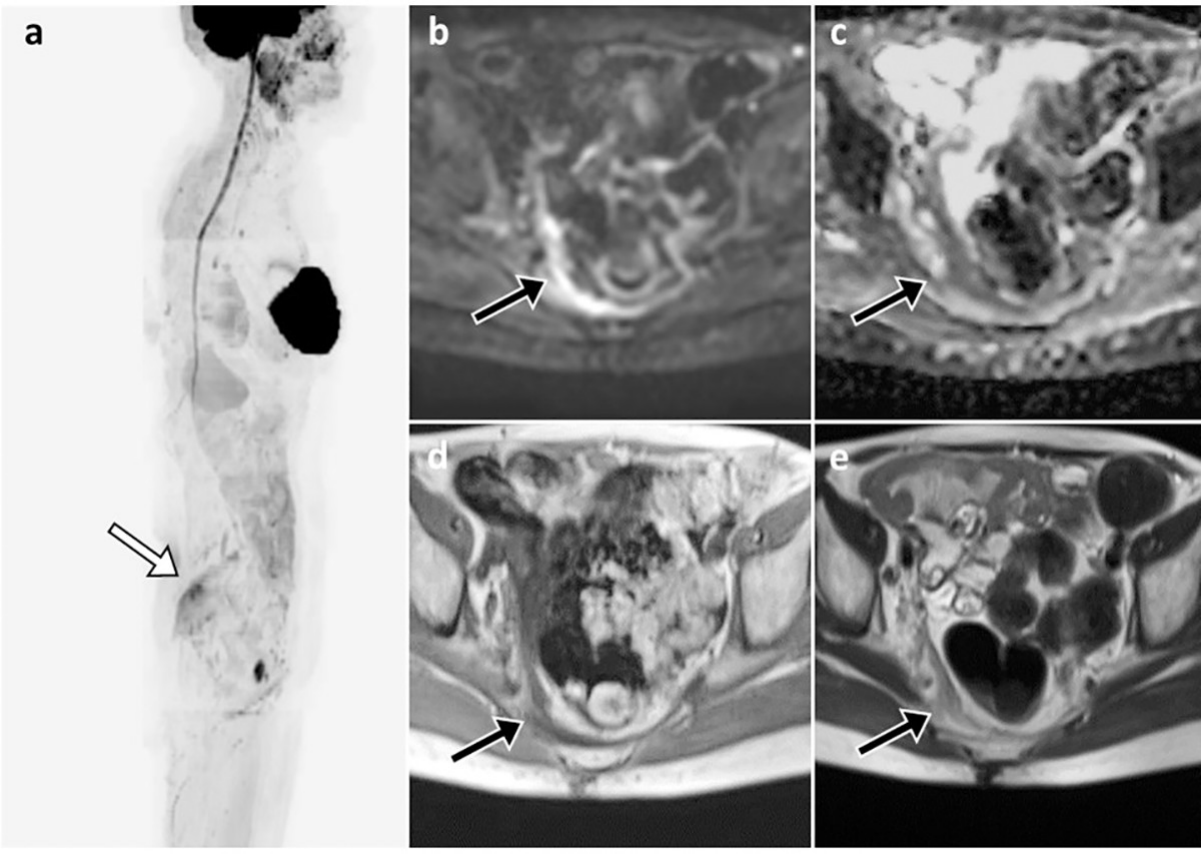
In the same patient of Fig 5, the paired WB-MRI showed thickening of the peritoneum in the right pelvis, which was hyper-intense in DWI b-900 sagittal MIP (a) and DWI axial b-900 images (arrow in b), with corresponding reduced ADC values (c). Axial T1 and T2 images confirmed the presence of a suspicious thickening of the right pelvic peritoneum and mesorectal fascia (arrows in d and e). No significant fluid collection can be seen. Other benign high signal intensity areas in the MIP image (a) include salivary glands, spinal cord, silicon breast implant, spleen, kidneys, small bowel and a bartholin’s gland cyst. The PD reported at WB-MRI determined a change in treatment from Letrozole to Fulvestrant.

The second most frequent AS on WB-MRI was peritoneum/retroperitoneum (Figs 5 and 6). MRI could be able to reveal peritoneal or retroperitoneal disease more confidently by means DWI, which can highlight small metastatic foci within normal tissues. In a study including 32 patients from Michielsen et al. WB-MRI (with DWI), PET/CT and CT were compared in the detection of peritoneal and retroperitoneal metastases of ovarian primary, with surgery as standard of reference. In this study WB-MRI was excellent in the detection of intraperitoneal disease, with sensitivity/specificity of 91% and 91%, compared to 65% and 82% for CT, and 52% and 85% for PET/CT, and was equivalent in the detection of retroperitoneal disease compared to PET/CT [32]. In another study including 26 patients from Fujii et al., the sensitivity and specificity of DWI for peritoneal carcinosis were 90% and 95.5%, respectively [33]. In our study, in 4 out of 6 WB-MRI with AS in peritoneum/retroperitoneum, the histology of the primary was ILC. This observation suggests that WB-MRI might be particularly suited for the assessment of ILC patients compared to CT and PET/CT. In ILC, metastatic spread to the gastrointestinal tract and to peritoneum/retroperitoneum is particularly frequent [34], and CT might be sometimes unable to detect these localizations, also because of its reduced sensitivity for small nodules (below 10 mm size) in the abdominal cavity [35] and in the retroperitoneum [36]. ILC, due to characteristic loss of E-cadherin adhesion protein [37], presents an un-aggregated growth [38], making metastases less measurable. PET/CT as well has a pitfall for small metastatic lesions below its spatial resolution, especially in the colon or in the small bowel where non-specific FDG uptake is often reported [39,40]. Moreover, the low FDG avidity of ILC [41] reduces the value of PET/CT for the detection of bone metastases in ILC [42].

Most of the AS reported by the control examination were in lymph nodes, being found in 3 PET/CT (sub-clavicular, hilar or mediastinal lymph nodes). This result might be related to the superior sensitivity and specificity of PET/CT (SE 98%,SP 83%) for lymph nodes compared to MRI in mediastinum (SE 80%, SP 75%) [43,44].

### Assessment of progressive disease and change in treatment

There were 17 episodes of PD reported only on WB-MRI and not reported on the control examination, accounting for 43% (17/40) of all cases of PD. Among these, 14 determined a change in treatment, accounting for 50% (14/28) of all changes after imaging evaluation. Evaluating these patients by means of WB-MRI resulted in a detection of PD that would have otherwise been recognized weeks or months later. This allowed the oncologists to interrupt ineffective treatments earlier, before the potential onset of acute complications of PD, such as SREs. The scope and methodology of our work are in line with a previously published work by Kosmin et.al.[45] that evaluates the impact of WB-MRI alongside CT-CAP on the management of MBC patients under systemic treatment. The study by Kosmin et.al. [45] showed that WB-MRI had an impact on treatment management in nearly 35% of all cases (where 16/46 of all changes in treatment were due to PD reported only on WB-MRI and not on CT-CAP). In the subgroup of WB-MRI paired to CT-CAP within our study, the impact of WB-MRI on treatment management was 70%. The greater impact of WB-MRI observed in our study could be due to different factors. In the study by Kosmin the time between WB-MRI and the control examination was below two weeks, therefore the greater interval between the examinations in our study could have allowed variations of the actual disease extent. A second reason for the different result could be the difference in population: 33% (19/58) of our examinations were performed on ILC patients while these formed only 12% of the population studied by Kosmin. We believe that the higher frequency of ILC patients in our study compared to the normal rate in breast cancer population (approximately 15%) [46] might have amplified the impact of WB-MRI on the detection of metastases. Thirdly, the presence of M0 patients in our cohort might have amplified the impact of WB-MRI on patient management, since the detection of metastases in these patients has a greater chance of leading to a change in treatment. Interestingly, there were no cases of PD reported only on the control examination, since all the AS reported on PET/CT occurred in episodes of PD involving also other anatomical sites, which were reported on both modalities.

There are some limitations to this study. Firstly, it was performed retrospectively without a new image analysis. This implies a certain degree of inhomogeneity between the original reports in terms of communication structure and level of detail, especially among the control examinations.We believe however that this kind of analysis is more relatable to true clinical practice. Secondly, the distance (median 27 days) between WB-MRI and the control examinations is quite high. It is important to note, however, that ours is not a perspective study, in which the schedule of imaging controls is fixed in the most convenient way. When oncologists in our institute program WB-MRI for an ABC patient, other imaging modalities such as CT or PET/CT are dismissed or sometimes interleaved at 3–6 months. This implies a subjective choice by the oncologist: indeed ESMO guidelines for ABC do not recommend a specific modality for follow-up [8]. These factors, combined with the waiting list for WB-MRI slots, motivated the quite high distance between WB-MRI and control examinations. Based on the findings of this study, we are currently planning a perspective study with a distance between WB-MRI and the control examination within seven days. Thirdly, our study does not address an eventual impact of WB-MRI on patient survival. This limitation is common to other imaging studies. Nevertheless, the earlier detection of PD and the subsequent changes in treatment might help delaying the onset of SREs and of other acute complications in metastatic patients, with a potential impact on quality of life and disability.

In conclusion, WB-MRI disclosed PD earlier than the control examination (CT-CAP or PET/CT) and it was responsible alone for 50% of all changes in treatment in our cohort. Larger and prospective studies are therefore encouraged to validate our observations in ABC patients.

## Supporting information

S1 Table Detailed results for all paired evaluations. Abbreviations: G = grade; ER = oestrogen receptor status; PG = progesterone receptor status; HER2 = expression of human epidermal growth factor receptor 2; M+ = metastatic status (TNM); S = site of disease; AS = additional site of disease; AS (PD) = additional site of disease that determined the outcome of PD; SD = stable disease; PD = progressive disease; PD (MRI only) = PD reported only on WB-MRI.
(XLSX)

## References

[1] Breast cancer survival statistics | Cancer Research UK [Internet]. [cited 2017 May 13]. Available from: http://www.cancerresearchuk.org/health-professional/cancer-statistics/statistics-by-cancer-type/breast-cancer/survival#heading-Three

[2] Sundquist M, Brudin L, Tejler G. Improved survival in metastatic breast cancer 1985–2016. The Breast [Internet]. 2017 2 [cited 2017 May 13];31:46–50. Available from: http://linkinghub.elsevier.com/retrieve/pii/S0960977616301874 10.1016/j.breast.2016.10.005 27810699

[3] National Collaborating Centre for Cancer (Great Britain). Advanced breast cancer: diagnosis and treatment: full guideline National Collaborating Centre for Cancer; 2009. 98 p.

[4] Coleman RE. Clinical Features of Metastatic Bone Disease and Risk of Skeletal Morbidity. Clin Cancer Res [Internet]. 2006 10 15 [cited 2017 Jun 25];12(20):6243s–6249s. Available from: http://www.ncbi.nlm.nih.gov/pubmed/17062708 17062708 10.1158/1078-0432.CCR-06-0931

[5] Kennecke H, Yerushalmi R, Woods R, Cheang MCU, Voduc D, Speers CH, et al Metastatic Behavior of Breast Cancer Subtypes. J Clin Oncol [Internet]. 2010 7 10 [cited 2017 Jun 14];28(20):3271–7. Available from: http://www.ncbi.nlm.nih.gov/pubmed/20498394 10.1200/JCO.2009.25.9820 20498394

[6] Body J-J, Quinn G, Talbot S, Booth E, Demonty G, Taylor A, et al Title: Systematic review and meta-analysis on the proportion of patients with breast cancer who develop bone metastases. Crit Rev Oncol [Internet]. 2017 [cited 2017 May 27]; Available from: http://dx.doi.org/10.1016/j.critrevonc.2017.04.008 28602171

[7] POCKETT RD, CASTELLANO D, MCEWAN P, OGLESBY A, BARBER BL, CHUNG K. The hospital burden of disease associated with bone metastases and skeletal-related events in patients with breast cancer, lung cancer, or prostate cancer in Spain. Eur J Cancer Care (Engl) [Internet]. 2010 11 [cited 2017 Jun 3];19(6):755–60. Available from: http://www.ncbi.nlm.nih.gov/pubmed/19708928 19708928 10.1111/j.1365-2354.2009.01135.x PMC3035821

[8] Cardoso F, Costa A, Senkus E, Aapro M, André F, Barrios CH, et al 3rd ESO–ESMO international consensus guidelines for Advanced Breast Cancer (ABC 3). The Breast. 2016; 10.1016/j.breast.2016.10.001 27927580

[9] Eisenhauer EA, Therasse P, Bogaerts J, Schwartz LH, Sargent D, Ford R, et al New response evaluation criteria in solid tumours: Revised RECIST guideline (version 1.1). Eur J Cancer [Internet]. 2009 1 [cited 2017 Jun 3];45(2):228–47. Available from: http://linkinghub.elsevier.com/retrieve/pii/S0959804908008733 10.1016/j.ejca.2008.10.026 19097774

[10] Hamaoka T, Costelloe CM, Madewell JE, Liu P, Berry DA, Islam R, et al Tumour response interpretation with new tumour response criteria vs the World Health Organisation criteria in patients with bone-only metastatic breast cancer. Br J Cancer [Internet]. 2010 2 16 [cited 2017 Jun 8];102(4):651–7. Available from: http://www.ncbi.nlm.nih.gov/pubmed/20104228 10.1038/sj.bjc.6605546 20104228 PMC2837571

[11] Lin NU, Thomssen C, Cardoso F, Cameron D, Cufer T, Fallowfield L, et al International guidelines for management of metastatic breast cancer (MBC) from the European School of Oncology (ESO)–MBC Task Force: Surveillance, staging, and evaluation of patients with early-stage and metastatic breast cancer. The Breast [Internet]. 2013 6 [cited 2017 Jun 3];22(3):203–10. Available from: http://www.ncbi.nlm.nih.gov/pubmed/23601761 10.1016/j.breast.2013.03.006 23601761

[12] Ulaner GA, Hyman DM, Lyashchenko SK, Lewis JS, Carrasquillo JA. 89Zr-Trastuzumab PET/CT for Detection of Human Epidermal Growth Factor Receptor 2–Positive Metastases in Patients With Human Epidermal Growth Factor Receptor 2–Negative Primary Breast Cancer. Clin Nucl Med [Internet]. 2017 12 [cited 2018 May 29];42(12):912–7. Available from: http://insights.ovid.com/crossref?an=00003072-201712000-00002 10.1097/RLU.0000000000001820 28872549 PMC5708879

[13] Ulaner GA, Goldman DA, Corben A, Lyashchenko SK, Gönen M, Lewis JS, et al Prospective Clinical Trial of ^18^ F-Fluciclovine PET/CT for Determining the Response to Neoadjuvant Therapy in Invasive Ductal and Invasive Lobular Breast Cancers. J Nucl Med [Internet]. 2017 7 [cited 2018 May 29];58(7):1037–42. Available from: http://jnm.snmjournals.org/lookup/doi/10.2967/jnumed.116.183335 27856630 10.2967/jnumed.116.183335 PMC6944156

[14] Ulaner GA, Goldman DA, Gonen M, Pham H, Castillo R, Lyashchenko SK, et al Initial Results of a Prospective Clinical Trial of 18F-Fluciclovine PET/CT in Newly Diagnosed Invasive Ductal and Invasive Lobular Breast Cancers. J Nucl Med [Internet]. 2016 9 1 [cited 2018 May 29];57(9):1350–6. Available from: http://jnm.snmjournals.org/cgi/doi/10.2967/jnumed.115.170456 26940766 10.2967/jnumed.115.170456

[15] Wibmer AG, Hricak H, Ulaner GA, Weber W. Trends in oncologic hybrid imaging. Eur J hybrid imaging [Internet]. 2018 [cited 2018 May 25];2(1):1 Available from: http://www.ncbi.nlm.nih.gov/pubmed/29782605 29782605 10.1186/s41824-017-0019-6 PMC5954767

[16] Catalano OA, Daye D, Signore A, Iannace C, Vangel M, Luongo A, et al Staging performance of whole-body DWI, PET/CT and PET/MRI in invasive ductal carcinoma of the breast. Int J Oncol [Internet]. 2017 7 [cited 2018 May 29];51(1):281–8. Available from: https://www.spandidos-publications.com/10.3892/ijo.2017.4012 28535000 10.3892/ijo.2017.4012

[17] Cho N, Im S-A, Cheon GJ, Park I-A, Lee K-H, Kim T-Y, et al Integrated 18F-FDG PET/MRI in breast cancer: early prediction of response to neoadjuvant chemotherapy. Eur J Nucl Med Mol Imaging [Internet]. 2018 3 4 [cited 2018 May 29];45(3):328–39. Available from: http://link.springer.com/10.1007/s00259-017-3849-3 29101445 10.1007/s00259-017-3849-3

[18] Takahara T, Imai Y, Yamashita T, Yasuda S, Nasu S, Van Cauteren M. Diffusion weighted whole body imaging with background body signal suppression (DWIBS): technical improvement using free breathing, STIR and high resolution 3D display. Radiat Med [Internet]. [cited 2017 5 27];22(4):275–82. Available from: http://www.ncbi.nlm.nih.gov/pubmed/15468951 15468951

[19] Kwee TC, Takahara T, Ochiai R, Katahira K, Van Cauteren M, Imai Y, et al Whole-body diffusion-weighted magnetic resonance imaging. Eur J Radiol [Internet]. 2009 6 [cited 2017 May 27];70(3):409–17. Available from: http://linkinghub.elsevier.com/retrieve/pii/S0720048X09001818 19403255 10.1016/j.ejrad.2009.03.054

[20] Padhani AR, Gogbashian A. Bony metastases: assessing response to therapy with whole-body diffusion MRI. Cancer Imaging [Internet]. 2011 10 3 [cited 2017 May 27];(1A):S129–45. Available from: https://www.ncbi.nlm.nih.gov/pmc/articles/PMC3266569/22185786 10.1102/1470-7330.2011.9034 PMC3266569

[21] Padhani AR, Makris A, Gall P, Collins DJ, Tunariu N, de Bono JS. Therapy monitoring of skeletal metastases with whole-body diffusion MRI. J Magn Reson Imaging [Internet]. 2014 5 [cited 2017 Jun 3];39(5):1049–78. Available from: http://www.ncbi.nlm.nih.gov/pubmed/24510426 10.1002/jmri.24548 24510426

[22] Yankeelov TE, Arlinghaus LR, Li X, Gore JC. The role of magnetic resonance imaging biomarkers in clinical trials of treatment response in cancer. Semin Oncol [Internet]. 2011 2 [cited 2017 May 27];38(1):16–25. Available from: http://linkinghub.elsevier.com/retrieve/pii/S0093775410002290 10.1053/j.seminoncol.2010.11.007 21362513 PMC3073543

[23] Chen L, Liu M, Bao J, Xia Y, Zhang J, Zhang L, et al The correlation between apparent diffusion coefficient and tumor cellularity in patients: a meta-analysis. PLoS One [Internet]. 2013 [cited 2018 Sep 5];8(11):e79008 Available from: http://www.ncbi.nlm.nih.gov/pubmed/24244402 10.1371/journal.pone.0079008 24244402 PMC3823989

[24] White NS, McDonald C, McDonald CR, Farid N, Kuperman J, Karow D, et al Diffusion-weighted imaging in cancer: physical foundations and applications of restriction spectrum imaging. Cancer Res [Internet]. 2014 9 1 [cited 2018 Sep 5];74(17):4638–52. Available from: http://www.ncbi.nlm.nih.gov/pubmed/25183788 10.1158/0008-5472.CAN-13-3534 25183788 PMC4155409

[25] Feuerlein S, Pauls S, Juchems MS, Stuber T, Hoffmann MHK, Brambs H-J, et al Pitfalls in Abdominal Diffusion-Weighted Imaging: How Predictive is Restricted Water Diffusion for Malignancy. Am J Roentgenol [Internet]. 2009 10 23 [cited 2018 May 30];193(4):1070–6. Available from: http://www.ajronline.org/doi/10.2214/AJR.08.2093 19770331 10.2214/AJR.08.2093

[26] Morone M, Bali MA, Tunariu N, Messiou C, Blackledge M, Grazioli L, et al Whole-Body MRI: Current Applications in Oncology. Am J Roentgenol [Internet]. 2017 12 5 [cited 2018 May 30];209(6):W336–49. Available from: http://www.ajronline.org/doi/10.2214/AJR.17.17984 28981354 10.2214/AJR.17.17984

[27] Dimopoulos MA, Hillengass J, Usmani S, Zamagni E, Lentzsch S, Davies FE, et al Role of Magnetic Resonance Imaging in the Management of Patients With Multiple Myeloma: A Consensus Statement. J Clin Oncol [Internet]. 2015 2 20 [cited 2017 Jun 3];33(6):657–64. Available from: http://www.ncbi.nlm.nih.gov/pubmed/25605835 10.1200/JCO.2014.57.9961 25605835

[28] Walker R, Barlogie B, Haessler J, Tricot G, Anaissie E, Shaughnessy JD, et al Magnetic Resonance Imaging in Multiple Myeloma: Diagnostic and Clinical Implications. J Clin Oncol [Internet]. 2007 3 20 [cited 2017 Jun 3];25(9):1121–8. Available from: http://www.ncbi.nlm.nih.gov/pubmed/17296972 10.1200/JCO.2006.08.5803 17296972

[29] Padhani AR, Lecouvet FE, Tunariu N, Koh D, Keyzer F De, Collins DJ, et al METastasis Reporting and Data System for Prostate Cancer: Practical Guidelines for Acquisition, Interpretation, and Reporting of Whole-body Magnetic Resonance Imaging-based Evaluations of Multiorgan Involvement in Advanced Prostate Cancer. Eur Urol. 2016;(0):1–12. 10.1016/j.eururo.2016.05.033 PMC5176005 27317091

[30] Shie P, Cardarelli R, Brandon D, Erdman W, Abdulrahim N. Meta-analysis: comparison of F-18 Fluorodeoxyglucose-positron emission tomography and bone scintigraphy in the detection of bone metastases in patients with breast cancer. Clin Nucl Med. 2008;33(2):97–101. 10.1097/RLU.0b013e31815f23b7 18209527

[31] Yang H-L, Liu T, Wang X-M, Xu Y, Deng S-M. Diagnosis of bone metastases: a meta-analysis comparing 18FDG PET, CT, MRI and bone scintigraphy. Eur Radiol [Internet]. 2011 12 2 [cited 2017 Jun 3];21(12):2604–17. Available from: http://www.ncbi.nlm.nih.gov/pubmed/21887484 10.1007/s00330-011-2221-4 21887484

[32] Michielsen K, Vergote I, Op de beeck K, Amant F, Leunen K, Moerman P, et al Whole-body MRI with diffusion-weighted sequence for staging of patients with suspected ovarian cancer: a clinical feasibility study in comparison to CT and FDG-PET/CT. Eur Radiol [Internet]. 2014 4 11 [cited 2017 Dec 12];24(4):889–901. Available from: http://link.springer.com/10.1007/s00330-013-3083-8 24322510 10.1007/s00330-013-3083-8

[33] Fujii S, Matsusue E, Kanasaki Y, Kanamori Y, Nakanishi J, Sugihara S, et al Detection of peritoneal dissemination in gynecological malignancy: evaluation by diffusion-weighted MR imaging. Eur Radiol [Internet]. 2008 1 14 [cited 2017 Jun 15];18(1):18–23. Available from: http://www.ncbi.nlm.nih.gov/pubmed/17701040 10.1007/s00330-007-0732-9 17701040

[34] Kwast ABG, Groothuis-Oudshoorn KCGM, Grandjean I, Ho VKY, Voogd AC, Menke-Pluymers MBE, et al. Histological type is not an independent prognostic factor for the risk pattern of breast cancer recurrences. Breast Cancer Res Treat [Internet]. 2012 8 19 [cited 2017 Jun 25];135(1):271–80. Available from: http://link.springer.com/10.1007/s10549-012-2160-z 22810087 10.1007/s10549-012-2160-z

[35] Coakley F V., Choi PH, Gougoutas CA, Pothuri B, Venkatraman E, Chi D, et al Peritoneal Metastases: Detection with Spiral CT in Patients with Ovarian Cancer. Radiology [Internet]. 2002 5 [cited 2017 Jun 15];223(2):495–9. Available from: http://www.ncbi.nlm.nih.gov/pubmed/11997559 10.1148/radiol.2232011081 11997559

[36] MURAKAMI M, MIYAMOTO T, IIDA T, TSUKADA H, WATANABE M, SHIDA M, et al Whole-body positron emission tomography and tumor marker CA125 for detection of recurrence in epithelial ovarian cancer. Int J Gynecol Cancer [Internet]. 2006 2 [cited 2017 Jun 15];16(S1):99–107. Available from: http://www.ncbi.nlm.nih.gov/pubmed/16515575 16515575 10.1111/j.1525-1438.2006.00471.x

[37] Singhai R, Patil VW, Jaiswal SR, Patil SD, Tayade MB, Patil A V. E-Cadherin as a diagnostic biomarker in breast cancer. N Am J Med Sci [Internet]. 2011 5 [cited 2017 Jun 25];3(5):227–33. Available from: http://www.ncbi.nlm.nih.gov/pubmed/22558599 10.4297/najms.2011.3227 22558599 PMC3337742

[38] Goldstein NS. Does the Level of E-Cadherin Expression Correlate With the Primary Breast Carcinoma Infiltration Pattern and Type of Systemic Metastases? Am J Clin Pathol [Internet]. 2002 9 1 [cited 2017 Jun 25];118(3):425–34. Available from: https://academic.oup.com/ajcp/article-lookup/doi/10.1309/JMRD-W08Y-6K8M-7AD8 12219785 10.1309/JMRD-W08Y-6K8M-7AD8

[39] Ahmad Sarji S. Physiological uptake in FDG PET simulating disease. Biomed Imaging Interv J [Internet]. 2006 10 [cited 2017 Jun 25];2(4):e59 Available from: http://www.ncbi.nlm.nih.gov/pubmed/21614339 10.2349/biij.2.4.e59 21614339 PMC3097820

[40] Prabhakar HB, Sahani D V., Fischman AJ, Mueller PR, Blake MA. Bowel Hot Spots at PET-CT. RadioGraphics [Internet]. 2007 1 1 [cited 2017 Jun 25];27(1):145–59. Available from: http://pubs.rsna.org/doi/10.1148/rg.271065080 17235004 10.1148/rg.271065080

[41] Bos R, van der Hoeven JJM, van der Wall E, van der Groep P, van Diest PJ, Comans EFI, et al Biologic Correlates of ^18^ Fluorodeoxyglucose Uptake in Human Breast Cancer Measured by Positron Emission Tomography. J Clin Oncol [Internet]. 2002 1 15 [cited 2017 Jun 25];20(2):379–87. Available from: http://www.ncbi.nlm.nih.gov/pubmed/11786564 10.1200/JCO.2002.20.2.379 11786564

[42] Dashevsky BZ, Goldman DA, Parsons M, Gönen M, Corben AD, Jochelson MS, et al Appearance of untreated bone metastases from breast cancer on FDG PET/CT: importance of histologic subtype. Eur J Nucl Med Mol Imaging [Internet]. 2015 10 14 [cited 2017 Jun 25];42(11):1666–73. Available from: http://link.springer.com/10.1007/s00259-015-3080-z 25971426 10.1007/s00259-015-3080-z PMC4558334

[43] Kim HY, Yi CA, Lee KS, Chung MJ, Kim YK, Choi BK, et al Nodal Metastasis in Non–Small Cell Lung Cancer: Accuracy of 3.0-T MR Imaging. Radiology [Internet]. 2008 2 [cited 2017 Jun 27];246(2):596–604. Available from: http://www.ncbi.nlm.nih.gov/pubmed/18056854 10.1148/radiol.2461061907 18056854

[44] Schmidt GP, Baur-Melnyk A, Herzog P, Schmid R, Tiling R, Schmidt M, et al High-resolution whole-body magnetic resonance image tumor staging with the use of parallel imaging versus dual-modality positron emission tomography-computed tomography: experience on a 32-channel system. Invest Radiol [Internet]. 2005 12 [cited 2017 Jun 27];40(12):743–53. Available from: http://www.ncbi.nlm.nih.gov/pubmed/16304476 16304476 10.1097/01.rli.0000185878.61270.b0

[45] Kosmin M, Makris A, Joshi P V., Ah-See M-L, Woolf D, Padhani AR. The addition of whole-body magnetic resonance imaging to body computerised tomography alters treatment decisions in patients with metastatic breast cancer. Eur J Cancer [Internet]. 2017 5 [cited 2017 Jun 25];77:109–16. Available from: http://www.ncbi.nlm.nih.gov/pubmed/28390297 10.1016/j.ejca.2017.03.001 28390297

[46] Li CI, Uribe DJ, Daling JR. Clinical characteristics of different histologic types of breast cancer. Br J Cancer [Internet]. 2005 10 20 [cited 2017 Dec 12];93(9):1046–52. Available from: http://www.nature.com/articles/6602787 10.1038/sj.bjc.6602787 16175185 PMC2361680

